# Through-Hole Spiral Microstrip Resonator for Inline Dielectric Characterization of Liquids

**DOI:** 10.3390/s26051544

**Published:** 2026-03-01

**Authors:** Laura Arruzzoli, Giovanni Gugliandolo, Ester Zirilli, Mariangela Latino, Alessandro Pistone, Giovanni Crupi, Nicola Donato

**Affiliations:** 1 BIOMORF Department, University of Messina, 98125 Messina, Italy; laura.arruzzoli@unime.it (L.A.);; 2 Department of Engineering, University of Messina, 98155 Messina, Italy; ester.zirilli@studenti.unime.it (E.Z.); alessandro.pistone@unime.it (A.P.);; 3 National Research Council, Institute for Chemical-Physical Processes, 98158 Messina, Italy

**Keywords:** microstrip resonator, sensors, inkjet printing, dielectric characterization, biocompatible materials, in line liquid analysis

## Abstract

This study addresses the inline dielectric characterization of liquids using a prototype planar resonant sensor with two capacitively coupled spirals, fabricated by inkjet printing on a Rogers RO4003C substrate. The device includes a central hole designed to host a sample vial or a pipe, enabling contactless characterization of liquid solutions, including biological samples. Experimental validation includes stylus profilometry and optical microscopy to verify the thickness, uniformity, and continuity of the conductive film, as well as scattering parameter measurements in the frequency range from 3.5 GHz to 4.0 GHz. The frequency response exhibits two distinct resonances; the corresponding resonance parameters for each mode (resonant frequency $f_{r}$, amplitude, and quality factor *Q*) were extracted through complex-domain fitting using Lorentzian profiles. The electrical characterization of the device was assessed as a function of the effective permittivity of water–ethanol test mixtures by varying the ethanol volume fraction. The proposed sensor showed a monotonic and nearly linear response to ethanol concentration, with frequency sensitivities of approximately 20 kHz/% and coefficients of determination up to $R^{2}=0.99$.

## 1. Introduction

Accurate characterization of the dielectric properties of liquids is pivotal in many application fields. In food quality control, measuring the permittivity of liquid foods can reveal contamination or changes in composition [1,2,3]. In the biomedical field, dielectric characterization plays an integral role, as the permittivity of biofluids (e.g., blood and urine) correlates with analyte concentrations (e.g., glucose or protein content) [4,5,6,7]. Traditionally, the measurement of complex permittivity can be performed using different conventional techniques, including cavity perturbation [8], waveguide transmission and reflection methods [9], and parallel-plate capacitor setups [10]. However, despite their high measurement accuracy, these conventional methods exhibit several drawbacks. They often require direct contact with the sample under test or large sample volumes, potentially altering or contaminating the sample. In addition, they typically operate in a destructive or invasive manner, as the collected sample is consumed during the measurement process. Moreover, the measurement setups are generally complex and expensive, with limited or no portability. These limitations motivate research into alternative methods for the dielectric characterization of liquids.

In such a context, microwave resonant sensors have emerged as a powerful alternative for the dielectric characterization of materials [6,11,12], including liquid samples [2,3,4,5]. Unlike traditional methods, resonant sensors operate at discrete frequencies and exhibit one or multiple resonances that shift in response to small changes in the surrounding permittivity. This resonant behavior results in high sensitivity to small variations in dielectric properties. Planar microwave resonators, in particular, offer several advantages: they are compact and low-cost (as they can be fabricated using standard PCB manufacturing techniques), easily integrated into electronic boards, and can be engineered to enable contactless measurements [13,14]. Because the electromagnetic fields of a resonator extend into the surrounding medium, a liquid under test placed in proximity to the resonator perturbs the resonance frequency and amplitude in proportion to the complex permittivity of the liquid [14]. By tracking these resonance shifts, it is possible to quantitatively determine the dielectric properties of the liquid [14].

In recent years, several types of planar sensors have been proposed for the dielectric characterization of liquids [2,3,4,5]. These include microstrip ring or split-ring resonators (SRRs) [15], their complementary counterparts (CSRRs) [14], as well as defected ground structures [16], spiral resonators [17], and fractal geometries [18]. All these approaches share the same working principle: the resonant frequency and quality factor change when a liquid sample perturbs the local electromagnetic field. An important design choice for liquid sensors concerns how the sample interacts with the resonator. Direct deposition of liquid onto the resonator surface can maximize field interaction, but it may contaminate the sensor and is impractical for continuous-flow measurements. For this reason, non-contact configurations have been proposed [13,14,17], in which the liquid is contained in a vial, a tube, or a microchannel that passes through or across the resonator. The electromagnetic fields penetrate the tube to probe the liquid, thereby avoiding direct contact between the resonator metallization and the fluid. This approach enables reusable sensors and facilitates inline measurements of flowing liquids in process streams or microfluidic chips.

In [19], a 2.4 GHz through-hole CSRR is proposed for liquid characterization. The resonator is aligned with a microstrip line such that a glass capillary carrying the liquid passes normal to the sensor surface. The electric field between the CSRR and the microstrip line is strongly perturbed by the sample, enabling contactless measurements. Similarly, in [14], the authors fabricated a low-cost multiple CSRR (MCSRR) on an FR-4 substrate operating in the 2.45 GHz band. A small glass capillary was inserted through the resonator, thus allowing contactless measurements. The proposed device exhibited resonance shifts of up to approximately 400 MHz and quality factor variations of up to 31 for dielectric constant values ranging from 3.5 to 79.0. The device enabled permittivity measurements of pure liquids and water–ethanol mixtures with errors below 5%. In [20], a triple-ring CSRR on an FR-4 substrate with a through-hole is presented. A polypropylene capillary tube is positioned in the central region of the CSRR, allowing inline flow of liquid samples. The resonator, operating at 2.5 GHz, demonstrated a frequency sensitivity of approximately 7.03 MHz per unit dielectric constant for the tested liquids (water, methanol, and ethanol). Notably, the non-contact nature of this design enables continuous monitoring of fluids. A compact dual-spiral band-stop resonator operating near 1.95 GHz with a central sensing cavity is presented in [17]. Although not flow-through, this architecture concentrates the electric field between the intercoupled spirals to maximize the perturbation per unit volume and combines the measured stop-band shift with a machine-learning model to regress the dielectric parameters of ethanol-water mixtures.

In this work, a planar microstrip resonator sensor with a through-hole is proposed for non-contact, inline characterization of liquids. The sensor consists of a dual-spiral planar resonant structure, similar to that presented in [17], into which a small tube is inserted vertically through a drilled via hole in the resonator. This configuration enables contactless liquid characterization, consistent with approaches reported in recent literature. Compared to the design proposed in [17], the resonator geometry has been updated by changing the feedline coupling and by tuning the geometry to support multiple resonant modes within a narrow bandwidth, with the aim of improving sensor reliability and enhancing metrological performance.

Compared to the design proposed in [17], the resonator geometry has been revised by modifying the feedline coupling and by tuning the layout to support multiple resonant modes within a narrow bandwidth. The main novelty of this work is not limited to the resonator geometry, but lies in a metrologically oriented sensing platform and readout strategy aimed at robust and repeatable parameter extraction from measured $S_{21}$ data. Accordingly, the proposed modifications are intended to improve sensor reliability and overall metrological performance, deliberately prioritizing robustness and repeatability of the measurement over maximum sensitivity. In [17], the feed lines are directly connected to the resonator, resulting in strong external coupling and a band-stop response. In sensing applications, strong coupling may reduce the loaded quality factor and thus degrade frequency estimation, particularly when the sensing metric is extracted from deep transmission minima, where noise can significantly affect the measurements. By introducing capacitive coupling between the feed lines and the resonator, the coupling is weakened, the loaded *Q* is increased, and resonance tracking becomes possible using narrow, well-defined peaks in $|S_{21}|$ at higher signal levels. Moreover, the geometry is tuned to support two closely spaced modes within a narrow band, enabling future self-calibrating readout strategies based on multi-mode measurements [21].

The microwave resonator is excited via microstrip feedlines, and its resonance frequencies are continuously monitored using a vector network analyzer. As the permittivity of the liquid changes, the resonant parameters are perturbed, enabling real-time liquid characterization. The proposed prototype was fabricated using inkjet printing technology on a Rogers RO4003C (Rogers Corporation, Chandler, AZ, USA) substrate and validated by inserting a sample vial into the resonator hole, filled with known mixtures of water and ethanol. Water–ethanol solutions with varying concentrations (0–100% ethanol by volume) were introduced into the sample vial, and the corresponding resonant parameters were measured. The results confirmed good sensor linearity ($R^{2}$ up to 0.97) and a sensitivity of approximately 32.8kHz/% ethanol. It is worth noting that the inline, flow-through design is particularly advantageous for bioanalytical and industrial processes, as it can be integrated into a fluidic pipeline to provide continuous measurements.

The remainder of the paper is organized as follows. Section 2 is devoted to materials and methods, detailing the prototype design and fabrication, the morphological characterization, the experimental setup for electrical characterization, and the methods used to extract the main figures of merit. Section 3 presents the electrical characterization of the sensor using water/ethanol mixtures and discusses the obtained results. Finally, Section 4 provides conclusions and outlines future perspectives of this research.

## 2. Materials and Methods

In this section, the resonant sensor proposed in this study is presented through a three-part description covering the design, fabrication and morphological characterization of the prototype and, finally, the measurement procedure used to evaluate its performance.

### 2.1. Prototype Design

The geometry of the resonant sensor is based on a dual-spiral planar resonant structure, separated by a central square region. This configuration is inspired by the design proposed in [17]. The proposed resonator includes a central hole, designed to allow the insertion of a tube containing a liquid solution, depending on the intended application. Although the liquid does not come into direct contact with the sensor, it influences the electromagnetic behavior of the device by modifying its effective permittivity $\varepsilon_{\mathrm{eff}}$, which in turn alters the frequency response of the scattering parameters. In particular, three main resonance parameters are affected: the resonant frequency, the peak amplitude, and the quality (Q−) factor. The electromagnetic characterization of the device was carried out by analyzing the scattering (S−) parameters.

In this research, the forward transmission coefficient $S_{21}$ was considered for the analysis of the sensor response, and the three resonant parameters were defined as follows:

- Resonant frequency: the frequency at which a maximum in the magnitude of $S_{21}$ occurs;
- Q-factor: defined as the ratio between the resonant frequency and the −3dB bandwidth;
- Amplitude: corresponds to the magnitude of $S_{21}$ at the resonant frequency and is measured in dB.

Electromagnetic simulations were performed in MATLAB^®^ R2025a using the PCB Antenna Toolbox from MathWorks^®^ (Natick, MA, USA). A three-dimensional model was implemented, including the RO4003C substrate with its relative permittivity, loss tangent, and thickness, together with the printed metallization. A parametric sweep was carried out on the spiral layout and on the feedline–resonator coupling regions with the objective of obtaining two resonant modes within the target frequency band, separated by approximately 300 MHz. This value represents a practical compromise between compact bandwidth and stable identification of two nearby modes using the adopted double-Lorentzian fitting procedure. In addition, the optimization aimed at maximizing resonance visibility by increasing both the peak amplitude and the quality factor of the resonant peaks observed in $|S_{21}|$. All geometric sweeps respected the fabrication constraints of the Voltera NOVA platform (Voltera, Waterloo, ON, Canada), including the minimum printable track width and spacing. Since the removable vial filled with water–ethanol mixtures is not directly supported in the adopted simulation workflow, the sensitivity to the liquid was evaluated experimentally and is discussed in Section 3.

The coupled-ring configuration makes it possible to concentrate the electric field in the central sensitive area, improving the response of the device to dielectric variations of the analyzed material [17]. The dual spiral resonant structure has been optimized through computer simulations. The feedlines were designed to guarantee a characteristic impedance $Z_{0}$ of 50 Ω, ensuring optimal coupling with the vector network analyzer (VNA).

**Figure 1:**
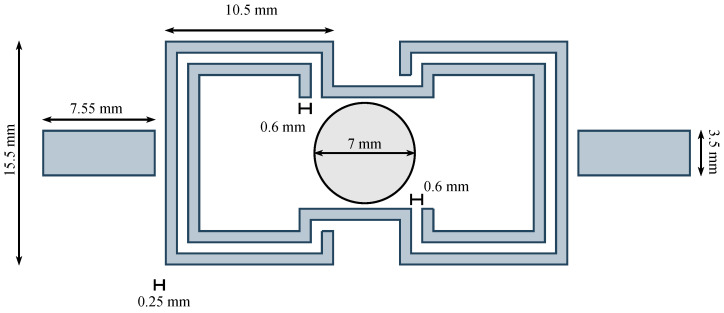
Technical illustration of the resonant sensor layout.

The chosen substrate is Rogers RO4003C, characterized by a dielectric constant of $\varepsilon_{r}=3.38$, a dissipation factor tanδ=0.0027, and a nominal thickness of h=1.52 mm. Figure 1 shows a schematic representation of the layout of the resonant sensor.

The planar resonator was fabricated using inkjet printing technology. This additive fabrication approach supports rapid prototyping of electronic boards and components, enabling fast design iterations at low cost. For this reason, it is increasingly adopted in the recent literature for antennas and microwave sensors [22,23], particularly when repeated geometry refinements are required. Conventional photolithography typically offers higher dimensional accuracy and lower conductor losses. Therefore, to ensure that the printed prototype is consistent with the intended design, a quantitative morphological characterization of the fabricated traces was included in this work to assess the fabrication accuracy.

The prototype was fabricated using the Voltera NOVA platform. This system is designed for rapid prototyping of circuits on both rigid and flexible substrates, and it can distribute conductive ink uniformly and continuously, even on surfaces that are not perfectly flat, with a resolution below 10 µm. For this deposition, the conductive ink Voltera Conductor FS0142 was used [24], a silver nanoparticle-based conductive formulation, applied through an extruder with a 150 µm nozzle diameter. The ink has a bulk resistivity lower than $6.0\times 10^{-6}\;\Omega \cdot \mathrm{cm}$ and a density of 3.72g/mL. The solid content is 81%, and the material provides good electrical performance even in the microwave range [24]. The printing was carried out on the Rogers RO4003C substrate, chosen for its stable dielectric properties and compatibility with microwave band applications. The laminate was cut to the desired final dimensions. To allow direct circuit printing, the top copper layer was peeled out from the ceramic substrate using a cutter, exposing the dielectric material, while the bottom metallic layer was kept intact, acting as a ground plane in the microstrip configuration.

After the printing process, the substrate was subjected to a thermal treatment in an oven at a nominal temperature of 150 °C for approximately 15 min. This step promoted solvent evaporation and sintering of the silver nanoparticles, resulting in a stable and homogeneous conductive matrix on the top surface. Following the curing process, a central hole with a nominal diameter of 7 mm was drilled, and the sensor surface was cleaned with isopropyl alcohol to remove any residual impurities. Finally, two SMA connectors were soldered to the feeding ports of the device to enable connection to the VNA.

**Figure 2:**
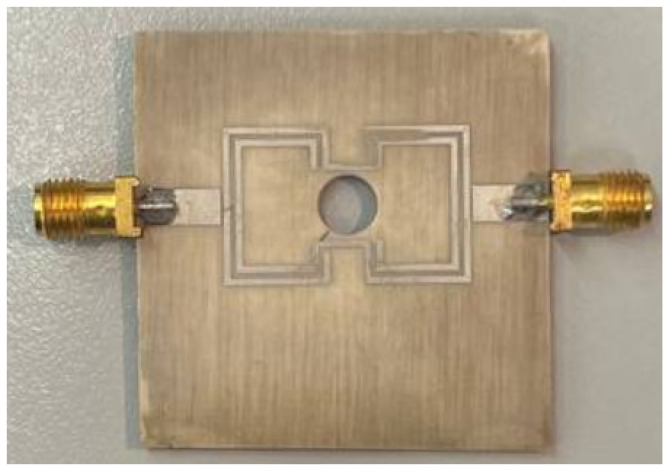
Fabricated resonant sensor prototype after integration of the central hole.

The fabricated device is shown in Figure 2.

### 2.2. Morphological Characterization

First, the device underwent morphological analysis to verify the quality of the conductive ink deposition and the geometric uniformity of the resonant structure. This analysis was performed using stylus profilometry with a Dektak XTL system (Bruker Corporation, Billerica, MA, USA), which provides submicrometric vertical resolution (up to 0.1 nm), a scanning area of 350 mm × 350 mm, and high repeatability and reproducibility [25]. This instrument enables the acquisition of three-dimensional surface maps and the extraction of thickness and width profiles of the conductive tracks, allowing the print quality to be correlated with the electrical performance of the sensor.

**Figure 3:**
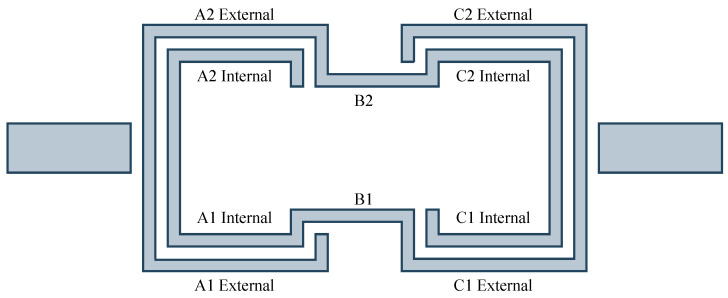
Regions of interest considered for profilometric characterization.

The measurements were managed using Vision64 software, which allowed different regions of interest on the device surface to be selected and automated profiling with micrometric steps to be performed. To assess uniformity across the entire surface, the sensor area was divided into ten regions, as illustrated in Figure 3:

- A1 external/A1 internal: portions of the lower left spiral;
- A2 external/A2 internal: portions of the upper left spiral;
- C1 external/C1 internal: portions of the lower right spiral;
- C2 external/C2 internal: portions of the upper right spiral;
- B1/B2: central portions delimiting the through-hole.

**Figure 4:**
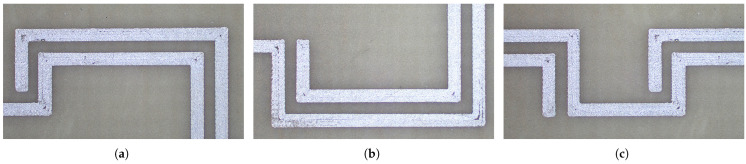
Optical microscope images acquired in three representative regions of the sensor: (**a**) C2 upper right, (**b**) C1 lower right, (**c**) B2 upper central area.

After defining the regions of interest, images were acquired using an optical microscope to perform a qualitative evaluation of the surface quality of the conductive film and to verify the absence of deposition defects or discontinuities in the tracks. The optical micrographs shown in Figure 4 illustrate, as representative examples, three of the analyzed areas: C2, C1, and B2, corresponding respectively to the upper right, lower right, and upper central regions of the resonator.

**Figure 5:**
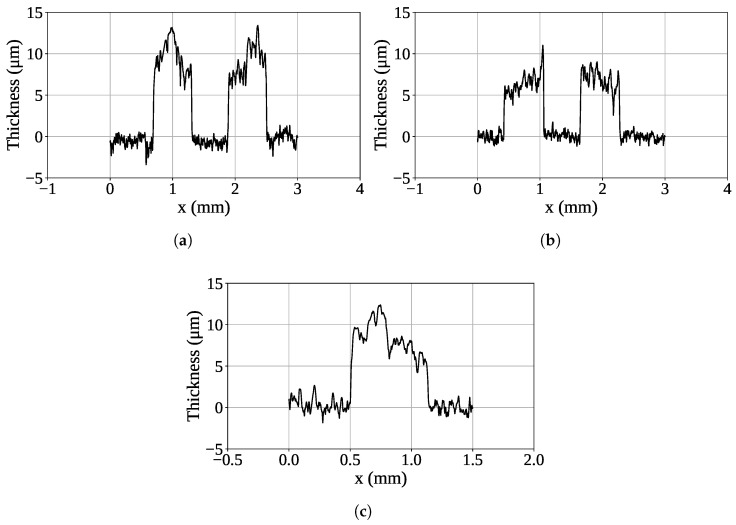
Stylus profilometer measurements acquired along the (**a**) C2, (**b**) C1, and (**c**) B2 sections of the sensor.

Following the optical inspection, a detailed profilometric analysis was performed on the selected regions to quantitatively evaluate the thickness of the conductive layer. Figure 5 reports the profilometric profiles acquired along the selected sections, where the vertical axis represents the measured film thickness and the horizontal axis represents the scan length in mm.

Statistical analysis of the data obtained from the profilometric measurements revealed an overall average thickness of 5.8 µm, with a global standard deviation of 2.4 µm. This result demonstrates the good quality of the printing process, as the measured thickness is consistent with the specifications declared by Voltera for the FS0142 ink, whose nominal printed thickness is approximately 7 µm. Overall, this indicates a sufficiently uniform deposition. However, a spatial analysis revealed slight variations between the upper and lower regions of the resonator. In the lower areas of the structure, reduced thickness values were observed compared to the upper regions, which exhibited thicker and more uniform films. This distribution suggests minor nonuniformities in ink flow during deposition, likely due to slight differences in substrate flatness or planarity. The morphological analysis was further extended to evaluate the average width of the conductive tracks, which confirmed the geometric consistency of the printed layout with respect to the CAD design. The measured average width was 0.60 mm, with a standard deviation of 0.12 mm, compared to the nominal value of 0.6 mm specified during the design phase. This agreement confirms the high accuracy of the printing process.

### 2.3. Electrical Characterization

**Figure 6:**
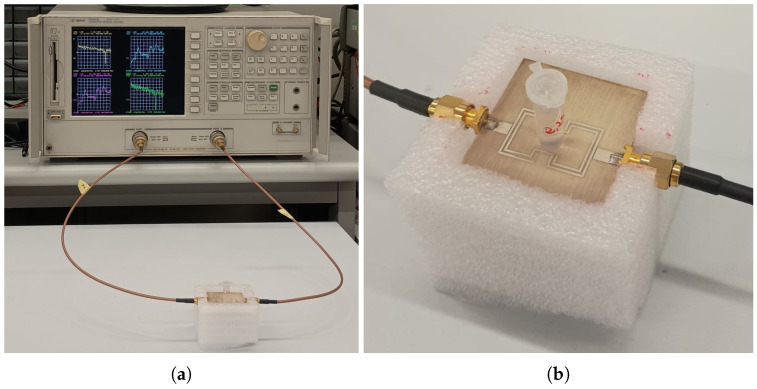
(**a**) Setup of the measurement system showing the two-port sensor connected to the VNA for in-line liquid characterization. (**b**) Close-up view of the water–ethanol mixture sample placed in the central cavity of the sensor, housed in an Eppendorf with a total capacity of 6 mL.

**Figure 7:**
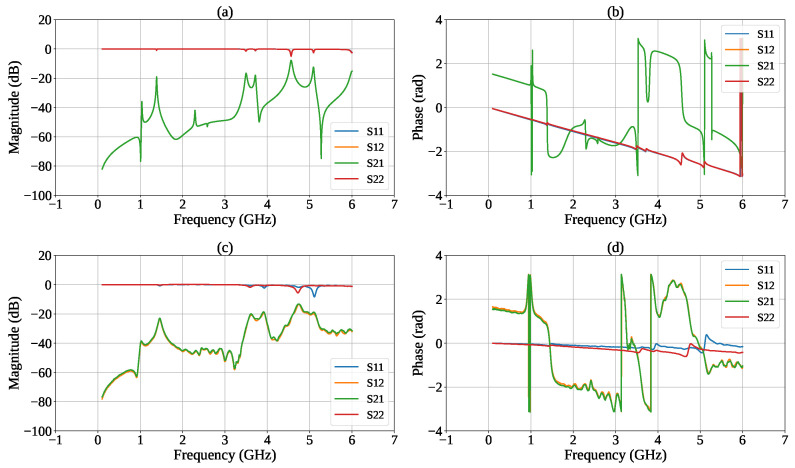
Simulated (**a**) magnitude and (**b**) phase and measured (**c**) magnitude and (**d**) of the resonator scattering parameters in the frequency range from 100 MHz to 6 GHz.

The electrical characterization of the sensor was performed using an Agilent 8753ES VNA (Agilent Technologies, Santa Clara, CA, USA) to evaluate the frequency response of the resonant structure and to analyze the influence of different dielectric constants of the liquid samples. The connection to the prototype was made using two coaxial cables, as shown in Figure 6a. Prior to the measurements, a full two-port Short-Open-Load-Through (SOLT) calibration was performed to establish the measurement reference planes at the device ports and to correct for systematic errors of the VNA measurement system. Measurements were carried out by connecting the sensor to the two ports of the VNA and acquiring the scattering parameters over the frequency range from 0.1 GHz to 6 GHz, with a resolution of 1601 points, a signal power of 10 dBm, and an intermediate frequency (IF) bandwidth of 300 Hz. Figure 7 reports the simulated and measured magnitude and phase of the S-parameters.

In this work, the parameter of interest was the forward transmission coefficient $S_{21}$, which was used to identify resonance conditions and quantify the sensor response as a function of the dielectric properties of the samples.

A preliminary characterization of the sensor was performed over the full 1–6 GHz range, revealing three pairs of resonant peaks. The first pair, located between 0.9 and 1.6 GHz, exhibited low sensitivity of approximately 1 kHz/%. The third pair, in the 4.6–5.3 GHz range, showed the highest sensitivity of about 30 kHz/% but was affected by significant signal degradation, which made the extraction of the sensing metric unreliable. Consequently, the second pair in the 3.5–4.0 GHz band was selected for detailed analysis, as it offered the best compromise between sensitivity (about 20 kHz/%) and spectral noise, enabling robust and reliable peak tracking.

For experimental validation, mixtures of water and ethanol at different volume fractions were used. Water, with a relative permittivity $\varepsilon_{r}=80.2$ [26], and ethanol, with $\varepsilon_{r}=25.16$ [26], span a wide range of $\varepsilon_{r}$ values, including those typical of biological fluids and aqueous solutions in the biomedical field, making the sensor potentially suitable for such applications. The solutions were prepared at five volume fractions (i.e., 100% H_2_O, 75% H_2_O–25% EtOH, 50% H_2_O–50% EtOH, 25% H_2_O–75% EtOH, and 100% EtOH) and were contained in a commercial 6 mL Eppendorf tube positioned in the central hole of the sensor (see Figure 6b).

**Figure 8:**
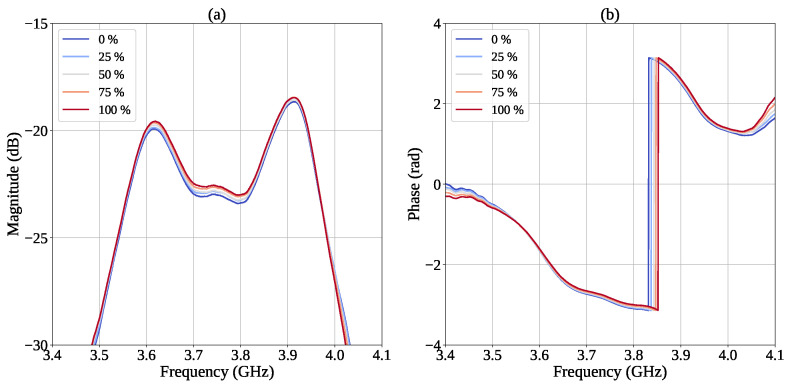
Measured (**a**) magnitude and (**b**) phase of $S_{21}$ at different ethanol volume fractions.

Once the sample was placed inside the central hole of the sensor, the scattering parameters were measured by connecting the device to the two ports of the VNA. Preliminary experimental tests indicated that the highest sensitivity is achieved in the frequency range between 3.5 GHz and 4.0 GHz, where two closely spaced resonant peaks in $|S_{21}|$ are observed, centered at approximately 3.6 GHz and 3.9 GHz. Accordingly, subsequent measurements were focused on this frequency band. For each mixture, the forward transmission coefficient $S_{21}$ was acquired. The magnitude and phase of the measured transmission coefficient as a function of frequency for all the tested ethanol volume fractions are reported in Figure 8.

To quantitatively evaluate the resonance parameters, namely the resonant frequencies, peak amplitudes, and Q-factors for each resonance, the experimental $S_{21}$ transmission data were processed using an analytical fitting procedure [21,27] based on the superposition of two complex Lorentzian functions, each corresponding to one of the resonant modes identified in the frequency response. This procedure was required because variations in the spectral response induced by changes in sample concentration were not easily distinguishable from the raw experimental data alone. Indeed, the two resonant peaks partially overlap in frequency, making direct and accurate estimation of the resonance parameters challenging. The use of a Lorentzian model allows the contributions of the individual modes to be separated, thereby improving the accuracy in the identification of the resonant frequencies and the determination of the associated quality factors.

The adopted mathematical model represents the complex transmission coefficient as the sum of two Lorentzian terms and a slowly varying background contribution:

(1) $$S_{21}\left(f\right)=\sum_{i=1}^{2}L_{i}\left(f\right)+B\left(f\right)$$

where each function $L_{i}\left(f\right)$ describes the response of the individual resonant mode according to the relationship:

(2) $$L_{i}\left(f\right)=\frac{a_{i}f}{f^{2}-w_{i}^{2}}$$

with complex coefficients $a_{i}$ and $w_{i}$. The complex frequency $w_{i}$ is defined as:

(3) $$w_{i}=f_{i}+jg_{i}$$

where $f_{i}$ represents the resonance frequency, and $g_{i}$ is related to the bandwidth at half power, from which the quality factor is derived:

(4) $$Q_{i}=\frac{f_{i}}{2g_{i}}$$

The term B(f) represents the background contribution and is modeled as a second order complex polynomial, useful for compensating for small mismatches or residual measurement effects:

(5) $$B\left(f\right)=\sum_{k=0}^{2}\left(a_{k}+ib_{k}\right)f^{k}$$

**Figure 9:**
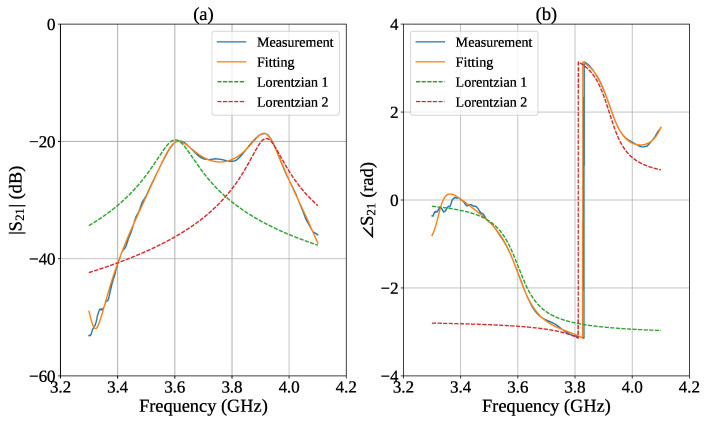
Measured and fitted $S_{21}$ curves for (**a**) magnitude and (**b**) phase, with the two Lorentzian functions obtained from the fitting process represented by dashed lines.

The fitting algorithm was implemented in Python 3.13 using the *lmfit* 1.3.3 library, which incorporates the Levenberg-Marquardt optimization algorithm. A complex-domain fitting approach was adopted, in which both the real and imaginary parts of $S_{21}$ were fitted simultaneously. Figure 9 shows the measured and fitted curves for both the magnitude and phase of $S_{21}$. The blue trace corresponds to the experimental data, while the orange line represents the overall fit obtained from the superposition of the Lorentzian components, which are shown in the figure as dashed lines.

**Figure 10:**
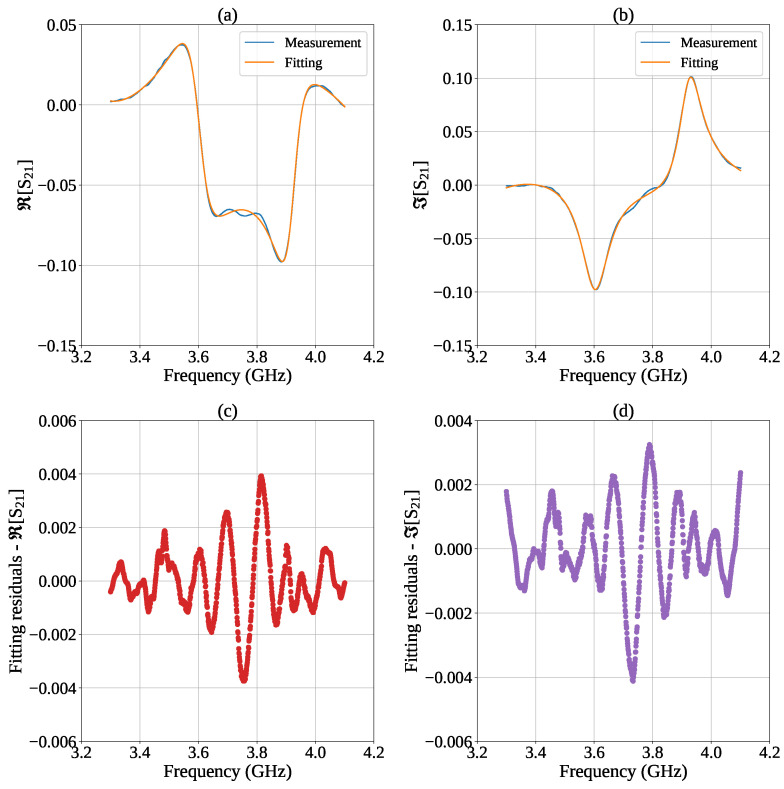
Measured and fitted $S_{21}$ curves for (**a**) real and (**b**) imaginary parts. (**c**,**d**) represent the corresponding fitting residuals.

The goodness of the fitting model was assessed through an analysis of the residuals, defined as the difference between the measured and fitted complex data. Figure 10 shows the real and imaginary residuals obtained after convergence of the fitting algorithm. Over the entire frequency range, the residuals exhibit random fluctuations centered around zero, and their amplitude remains mostly below $4\times 10^{-3}$, confirming that the adopted analytical formulation accurately captures the physical response of the device.

From the analytical expressions obtained through the fitting process, the main resonant parameters of the sensor can be determined. For each Lorentzian component, the resonant frequency $f_{i}$, the corresponding amplitude $|L_{i}\left(f_{i}\right)|$, and the quality factor $Q_{i}$ are directly extracted and discussed in Section 3.

## 3. Results

**Figure 11:**
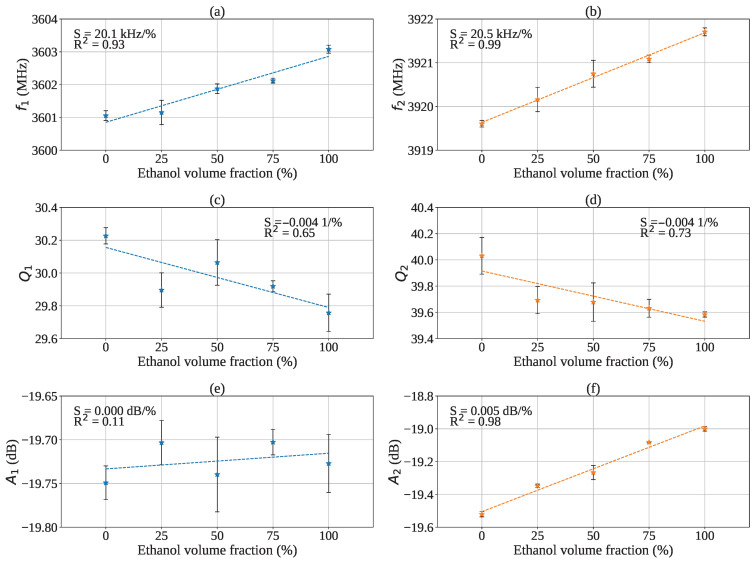
Extracted resonant parameters of the two resonance modes as a function of ethanol volume fraction. The first row reports the resonant frequencies: (**a**) $f_{1}$ and (**b**) $f_{2}$. The second row reports the corresponding quality factors: (**c**) $Q_{1}$ and (**d**) $Q_{2}$. The third row shows the amplitudes: (**e**) $A_{1}$ and (**f**) $A_{2}$. A linear regression (dashed line) is applied to each dataset; the slope (sensitivity) and the coefficient of determination $R^{2}$ are reported within each plot.

The two resonant modes of the device were identified through the fitting procedure applied to the measured data, and their resonant parameters were extracted for each ethanol volume fraction to assess the sensor response. The trends of the extracted parameters as a function of ethanol concentration are reported in Figure 11.

Figure 11a,b show the resonant frequencies $f_{1}$ and $f_{2}$ as a function of ethanol volume fraction. Both resonances exhibit a clear and monotonic shift toward higher frequencies as the ethanol content increases, which is consistent with the expected reduction of the effective permittivity of the mixture when moving from water-rich to ethanol-rich solutions. A linear regression yields sensitivities of 20.1kHz/% ($R^{2}=0.93$) for $f_{1}$ and 20.5kHz/% ($R^{2}=0.99$) for $f_{2}$. Although the two modes show comparable sensitivity, the second mode exhibits a higher linearity and is therefore a stronger candidate as the primary sensing parameter.

The corresponding quality factors are reported in Figure 11c,d. Both $Q_{1}$ and $Q_{2}$ show a modest decrease with increasing ethanol volume fraction, with slopes of −0.0041/% and coefficients of determination $R^{2}=0.65$ and $R^{2}=0.73$, respectively. This behavior is consistent with expectations, since ethanol exhibits a higher loss tangent than water [28]. As the ethanol volume fraction increases, the loss tangent of the mixture increases, resulting in higher overall resonator losses and a reduction of the *Q*-factor. Overall, the observed variations in *Q* are limited compared to the frequency shifts.

The amplitude trends are depicted in Figure 11e,f. The amplitude $A_{1}$ remains nearly constant across the tested concentrations, indicating a negligible dependence on ethanol fraction for the first mode. In contrast, $A_{2}$ exhibits a clear monotonic increase with ethanol content, with a sensitivity of 0.005dB/% and $R^{2}=0.98$, suggesting that the second mode amplitude can be used as an additional indicator of composition changes.

The data reported in the Figure 11 show a certain degree of dispersion, whose extent was evaluated through repeatability and reproducibility measurements, in order to quantify the combined effect of the main sources of uncertainty that may influence the resonant parameters extracted from the experimental curves. The considered uncertainty sources contributing to the variability of the measurements include misalignments in the sample positioning within the central hole of the resonator, variations in the effective concentration between samples due to small differences in preparation or in the amount of mixture inserted, instrumental uncertainties related to the VNA, and approximations of the Lorentzian fitting model, whose accuracy may decrease when the resonances are very close to each other or partially overlapped. The sensor response was therefore evaluated by performing a series of repeated measurements under nominally identical conditions, separated by short time intervals. For each acquisition, the sample was removed and repositioned inside the resonator hole, thus simulating realistic operating conditions and including possible effects of alignment or contact. From each measurement, the resonant parameters ($f_{i}$, $A_{i}$, $Q_{i}$) related to the two modes of the device were extracted, and the corresponding standard deviations were calculated, resulting below approximately 0.6 MHz for the resonant frequencies, 0.03 dB for the peak amplitudes, and 2.2 for the Q-factors.

## 4. Conclusions

In this work, a planar microstrip resonant sensor fabricated by inkjet printing on a Rogers RO4003C substrate was presented. The device incorporates a through-hole to accommodate a vial or a tube and is intended for inline, contactless dielectric characterization of liquids. The inkjet-printing fabrication quality was assessed by stylus profilometry and optical microscopy, which confirmed good uniformity of the printed conductive film and geometric fidelity to the CAD layout, in agreement with the conductive ink specifications. The adoption of capacitive feedline coupling enables peak-based tracking with increased loaded *Q* and improved robustness of resonant-parameter extraction. The presence of two closely spaced modes in a narrow band provides a practical basis for future self-calibrating strategies using multi-mode measurements. The sensor response was investigated through measurements of the forward transmission coefficient $S_{21}$. Two closely spaced resonant modes were identified in the considered frequency band, and their resonant parameters were quantified via complex-domain fitting using the superposition of two Lorentzian profiles with an additional slowly varying background term. Experimental validation was conducted using water–ethanol mixtures at different volume fractions, with the effective permittivity of the solutions used as the reference quantity. The resonances exhibited a monotonic and nearly linear dependence on the ethanol volume fraction with a sensitivity of about 20 kHz/%. For completeness, Table 1 provides a comparison with similar planar microwave sensors for water–ethanol mixtures reported in the literature. While the achieved sensitivity is moderate, the proposed platform is designed to prioritize measurement robustness, ensuring stable and repeatable extraction of resonant parameters from $S_{21}$ data, which is critical for inline liquid characterization in practical workflows.

Future developments will focus on optimizing the sample holder and its mechanical fixture to minimize misalignment and parasitic effects, and on extending the experimental campaign to flowing liquids by assessing potential influences of flow rate across the sensing region. In addition, future work will investigate implementations of the proposed design on thinner or flexible substrates [30,31,32]. Such platforms may facilitate improved conformability, potentially enhancing the electromagnetic coupling with the vial, tube, or pipe containing the liquid under test; however, transferring the present layout to thinner or flexible substrates will require a dedicated geometry optimization. Moreover, the characterization procedure will be extended to retrieve complex permittivity, including dielectric losses, with the goal of increasing the applicability of the proposed approach in real-world scenarios.

## Figures and Tables

**Table 1:** Comparison of planar microwave resonators for water–ethanol mixture sensing.

Reference	Resonator Type	Frequency (GHz)	Sensitivity	Multi-Mode Operation	Inline Configuration
[17]	Coupled spiral resonators	1.95	2.97 MHz/%	No	No
[29]	Coupled ring resonators	2–3	188 kHz/%	Yes	No
[19]	Complementary split-ring resonator	2.3	∼0.45 MHz/%	No	Yes
[20]	Complementary split-ring resonator	2.1–2.3	∼2 MHz/%	No	No
[30]	Split-ring resonators (RO4003C)	0.5–2.5	∼5 MHz/%	No	Yes
This work	Coupled spiral resonators	3.5–4.0	20.5 kHz/%	Yes	Yes

## Data Availability

No new data were created or analyzed in this study.
